# Effects of maxillary expansion on hearing and voice function in non-cleft lip palate and cleft lip palate patients with transverse maxillary deficiency: a multicentric randomized controlled trial

**DOI:** 10.1016/j.bjorl.2019.09.010

**Published:** 2019-11-02

**Authors:** Harpreet Singh, Raj Kumar Maurya, Poonam Sharma, Pranav Kapoor, Tanmay Mittal, Mansi Atri

**Affiliations:** a ESIC Dental College and Hospital, Department of Orthodontics and Dentofacial Orthopedics, Delhi, India; b Army Dental Centre (Research & Referral), Department of Orthodontics and Dentofacial Orthopedics, New Delhi, India

**Keywords:** Cleft lip, Cleft palate, Hearing loss, Palatal expansion technique, Voice quality

## Abstract

**Introduction:**

The association between the treatment of transverse maxillary deﬁciency and the recovery of hearing and voice functions has gained attention in recent years.

**Objective:**

This prospective controlled trial aimed to evaluate the effects of rapid maxillary expansion on hearing and voice function in children with non-cleft lip palate and bilateral cleft lip palate with transverse maxillary deficiency

**Methods:**

53 patients (26 non-cleft and 27 bilateral cleft lip palate; mean age, 11.1 ± 1.8 years) requiring rapid maxillary expansion for correction of narrow maxillary arches were recruited for this trial. Eight sub-groups were established based on the degree of hearing loss. Pure-tone audiometric and tympanometric records were taken for each subject at four different time periods. The first records were taken before rapid maxillary expansion (T0), the second after expansion (T1) (mean, 0.8 months), the third after three months (T2) (mean, 3 months) and the fourth at the end of retention period (T3) (mean, 6 months). ANOVA and Tukey HSD post-hoc tests were used for data analysis. Additionally, voice analysis was done using an updated PRAAT software program in a computerized speech lab at T0 and T2. A paired-sample*t*-test was used for comparisons of mean values of T0 and T2 voice parameters within both groups.

**Results:**

Rapid maxillary expansion treatment produced a significant increase in the hearing levels and middle ear volumes of all non-cleft and bilateral cleft lip palate patients with normal hearing levels and with mild conductive hearing loss, during the T0–T1, T1–T2, T0–T2, and T0–T3 observation periods (p < 0.05). The significant increase was observed in right middle ear volumes during the T0–T1, T0–T2 and T0–T3 periods in non-cleft patients with moderate hearing loss. For voice analysis, significant differences were observed only between the T0 and T2 mean fundamental frequency (F0) and jitter percentage (p < 0.05) in the non-cleft group. In the cleft group, no significant differences were observed for any voice parameter between the T0 and T2 periods.

**Conclusion:**

Correction of the palatal anatomy by rapid maxillary expansion therapy has a beneficial effect on both improvements in hearing and normal function of the middle ear in both non-cleft and bilateral cleft lip palate patients. Similarly, rapid maxillary expansion significantly influences voice quality in non-cleft patients, with no significant effect in BCLP patients.

## Introduction

Transverse maxillary deficiencies represent a significant component of all malocclusions among both cleft lip palate and non-cleft lip palate orthodontic patients. Mouth breathing, digit and pacifier sucking, and atypical swallowing have been ascribed as the myriad causative factors of maxillary width deficiency, often resulting in detrimental effects on the dentofacial pattern.^1^

Estimates from the World Health Organization reveal that hearing loss is a globally prevalent public health problem with a sizeable number of populations (approx. 42 million people) more than three years of age being affected by it. Approximately, 28–38% of the children are affected by middle ear disorders with far-reaching adverse impacts on their psychosocial, esthetic, orofacial, academic and overall development.^2^, ^3^ Conductive Hearing Loss (CHL) is one of the common hearing disorders characterized by elevated air-conduction thresholds, as assessed by the pure-tone audiometry.^4^ A possible connection between CHL and maxillary constriction has been widely reported in the literature.^5^ Fingeroth^6^ attributed the genesis of decreased nasal permeability with mouth breathing to maxillary deficiency, which in turn provides a conducive environment for the development of CHL. Pathologic changes in the middle ear can also occur due to impaired Eustachian Tube (ET) functions, thus leading to hearing loss and/or other complications such as otitis media.^5^

Patients with Cleft Lip and Palate (CLP) usually encounter a multitude of lifelong challenges due to the constrained growth of the upper jaw; and frequently manifest speech, orthodontic, hearing and dental problems.^7^ Approximately 50–60% of individuals with CLP also exhibit abnormal middle ear status with a fluctuating and mild to moderate conductive hearing loss.^8^ Secretory otitis media occurring due to anatomical and functional malformations in the ET and in the velopharyngeal sphincter region is a frequent hearing-related alteration in such patients.^7^

The anatomical and physiological integrity of the hearing system exhibits a strong interrelationship with the acquisition and development of verbal language and speech.^7^ The deviant speech production in CLP is characterized by retracted articulation and hypernasality. Myriad predisposing risk factors for nasal speech in CLP patients include recurrent middle ear infections, neuromuscular disturbances, cerebral malformations, anatomical variations of the palate and velopharyngeal failure.^9^, ^10^ Also, the transverse maxillary discrepancy coupled with severely affected structure of the vocal tract can impact the oral, nasal, or pharyngeal areas vocal quality, which can be worsened due to CLP and surgeries associated with it.^11^ Maxillary expansion has been shown to contribute to improved nasal breathing, masticatory function, facilitation of future permanent tooth eruption, and enhanced aesthetics leading to greater self-esteem in CLP cases.

Substantial changes produced by Rapid Maxillary Expansion (RME) may change the oral, nasal, and pharyngeal tissue form, thereby contributing to improved respiration and correction of a dental cross-bite. In growing patients with CHL and associated maxillary deficiency, the orthopedic effect of RME may aid in improving hearing owing to a restoration of normal functioning of the pharyngeal ostia of the Eustachian tubes.^5^, ^12^, ^13^, ^14^, ^15^, ^16^

Although the association and correlation between the treatment of transverse maxillary constriction and the unexpected therapeutic recovery of auditory functions have been widely documented in the literature, a recent systematic review by Fagundes et al.^17^ stressed the need for more controlled and randomized trials to reach a more reliable conclusion. A few studies have also examined the effects of RME therapy on voice function in non-cleft patients.^18^, ^19^, ^20^

However, to the best of our knowledge, no studies have been performed that investigated the interventional effect of maxillary expansion on the improvement of both speech and hearing functions among non-cleft lip palate and cleft lip palate patients presenting with varying degree of hearing ability patterns. Thus, this prospective controlled trial aimed to evaluate the effects of RME among age-matched non-cleft patients and non-syndromic Bilateral Cleft Lip Palate (BCLP) patients requiring maxillary expansion as one of the treatment modalities for adjunctive correction or improvement of hearing and voice function.

## Materials and methods

The present study was conducted as an Inter-Hospital research project involving two University Centers. Approval for the study was obtained from the Institutional Ethical Committee vide Appx ‘A’ to O/o Institutional Permission letter nº 15965/56th; and the present clinical study was registered at the Institutional Health Care Registry, Research and Referral Center, vide ADC/Pers/RKM/2017 dated 11 Feb 2017. Written informed consent was obtained from the parents of all patients after explaining the details of the study. Based on the significance level of alpha 0.05 and at least 80 percent power with an allowable error of 15%, 53 patients within the age group of 9–13 years (mean age, 11.1 ± 1.8 years) were recruited for the study. The patients were divided into two groups: Group I consisted of 26 non-cleft patients (11 females, 15 males) requiring maxillary expansion for correction of transverse maxillary constriction; and Group II comprised 27 BCLP patients (13 females, 14 males) requiring maxillary expansion for correction of maxillary constriction and arch collapse.

Patients enrolled for this prospective study met the following inclusion criteria in cleft groups: (1) Presence of transverse maxillary deficiency; (2) bilateral posterior cross-bite, (3) lip and palatal repair in early childhood only with no subsequent surgical interventions, (4) no history of previous orthodontic treatment, (5) high degree of cooperation. The inclusion criteria for the noncleft control group were: (1) Angle’s Class I occlusion with bilateral posterior crossbite, (2) no history of previous orthodontic treatment and (3) internally motivated and cooperative. Exclusion criteria were as follows for both groups: (1) History of orthodontic treatment, (2) history of nasal or pharyngeal surgery, (3) presence of nasorespiratory and allergic symptoms (3) presence of systemic disease; (4) craniofacial anomalies and syndromic cases, and (5) presence of cerumen, tympanic membrane perforation and/or other possible alterations as confirmed by otoscopic examination.

**Figure 1:**
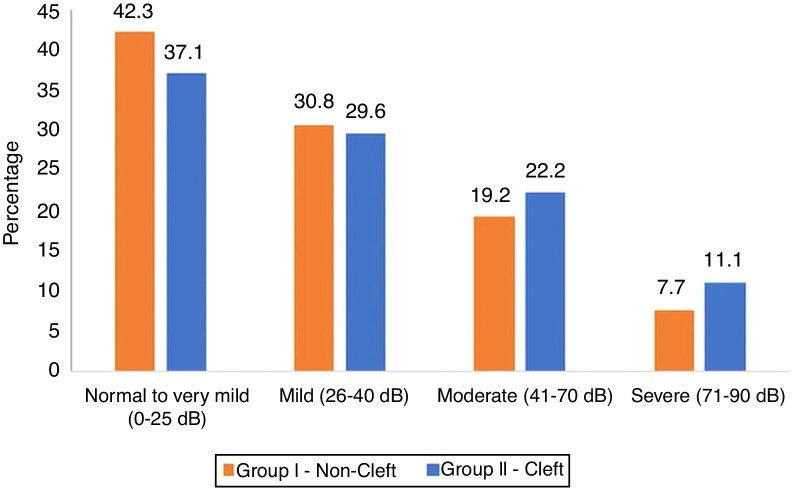
Histogram depicting percentage distribution of patients based on hearing abilities.

Based on the classification of degree of hearing loss,^21^ patients in non-cleft group i.e. Group I and cleft group i.e. Group II were sub-grouped as having: normal hearing levels to very mild hearing loss (Ia, IIa), mild hearing loss (Ib, IIb), moderate hearing loss (Ic, IIc), and severe hearing loss (Id, IId)). Percentage frequencies of subjects with a varying degree of hearing loss are shown in Fig. 1. Hearing loss in patients was purely a conductive and not a sensorineural or mixed one, as demonstrated by comparative measurements of air- and bone-conduction thresholds from pure-tone audiograms. Greater hearing loss was observed for frequencies between 250–1000 Hz as compared to frequencies between 1000–2000 Hz. Some patients with mild hearing loss were unaware of their hearing disabilities.

**Figure 2:**
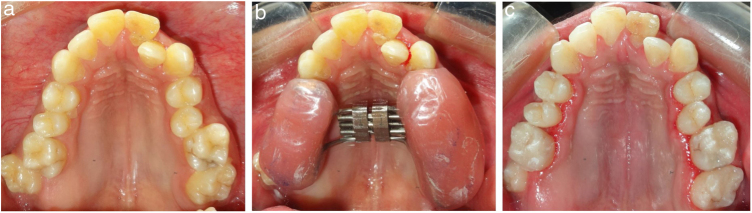
(a) Pretreatment maxillary occlusal view showing constricted maxillary arch in a non-cleft patient, (b) Bonded Hyrax assembly in a non-cleft patient, and (c) After completion of RME.

**Figure 3:**
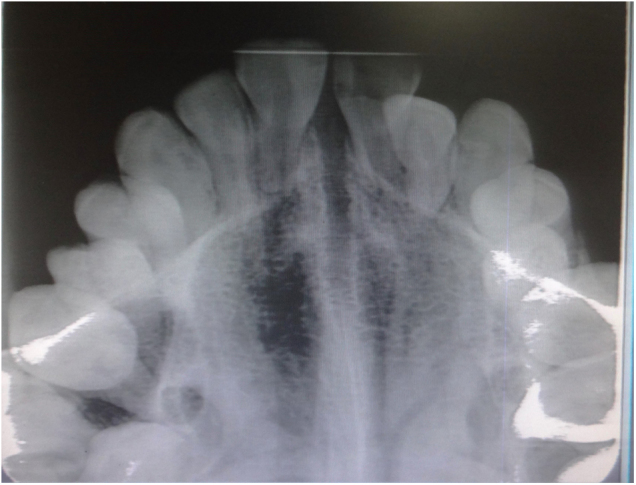
Occlusal view radiograph showing fan-shaped opening of the midpalatal suture in the same patient.

**Figure 4:**
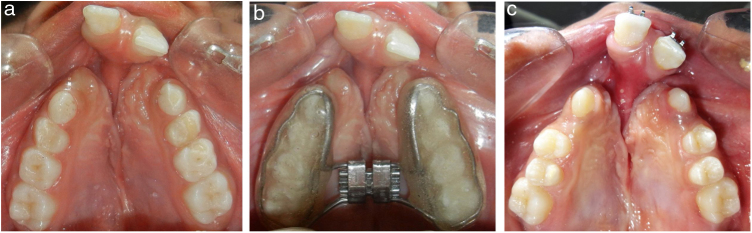
(a) Pretreatment maxillary occlusal view showing the upper arch collapse in bilateral cleft lip palate patient, (b) Bonded Hyrax assembly at the initiation of RME in the same patient and (c) Maxillary occlusal view after completion of RME.

Before the commencement of the RME procedure, the hearing levels of each patient were evaluated by an otorhinolaryngologist by means of pure-tone audiograms and tympanograms. RME was performed using bonded Hyrax expander (9 mm; Leonne, Italy), activated twice a day, one turn in the morning and one in the evening (0.5 mm/day), for 7–14 days until necessary expansion for each subject was accomplished with complete elimination of posterior crossbite (Figs. 2a–c , 3 and 4a–c). Thereafter, the screw was locked with a double ligature tie, and the expander served as a passive retainer for the next 3 months to allow for bone formation at the suture level and prevent relapse of the expansion space in the incisor region. After 3 months, the expander was removed and a rigid transpalatal arch with the extension throughout anterior teeth was inserted and used for 6 months, at which time the mineralization of the suture was completed.

Following the standardized protocols proposed by Munhoz et al.,^22^ all patients underwent the audiometric and tympanometry assessment tests at four different periods: T0 – before RME; T1 – at the end of RME (mean, 0.8 months); T2 ‒ 3 months after RME; and T3 – at the end of retention period of 6 months, in a sound-treated booth. All the assessments were done using properly calibrated interacoustic Maico MA 53 audiometer (Guymark, United Kingdom) with TDH-39 headphone and Grason-Stadler Middle Ear Analyzer version 2 Impedanciometer (Minnesota, USA), in a sound-treated booth. After the evaluation of all audiometric records by an otolaryngologist, the pure-tone thresholds at four speech frequencies of 250, 500, 1000 and 2000 Hz, were obtained separately for each ear. Tympanometric records were also used to determine the static compliance values and middle ear volumes. No anti-inﬂammatory drugs, antibiotics, or serous ﬂuidifying medicines were prescribed during treatment.

Acoustic voice samples were recorded from all patients at T0 i.e. before RME, and at T2 i.e. at the end of 3 months stabilization phase, without the appliance in the mouth. All voice samples were recorded in a quiet room using a high-quality unidirectional condenser microphone brand Shure, model SM-58, on a desktop computer (Dell 4, 3.2 GHz, 512 MB RAM). The microphone was positioned at a constant 10 cm distance from the mouth in a 45° to 90° off-axis position.^23^ In accordance with the technique of Boersma and Weenink,^24^ the manoeuvre for speech recording was as follows: At the outset, an initial rest period of at least 10 min was allowed for each subject. Thereafter, the subjects were instructed to phonate the vowel /a/ three times for at least 5 s in the comfortable sitting position, after a deep inspiration. The three recorded voice samples were relayed directly to the computer; and after choosing the highest-quality data from three recorded samples, the middle 3 s part was edited and analyzed with the updated PRAAT^24^ software program to reduce variability.

The hearing records (audiograms and tympanograms) and voice recordings were blocked and randomized for assessment by well-trained evaluator. This helped ensure blinding, thereby eliminating the influence of cognitive bias on the results in the assessments.

Following acoustic parameters were analyzed and compared between T0 and T2:

Mean fundamental frequency (F0; Hz): representing the number of vibrations of the vocal fold per second;

Jitter percentage (pitch perturbation): representing short-term (cycle-to-cycle) deviation in the fundamental frequency of a signal/deviation from true periodicity of a presumably periodic signal^25^;

Shimmer percentage (amplitude perturbation): representing the variability of the peak-to-peak amplitude between adjacent cycles of vocal fold vibrations^26^;

Harmonic-to-noise ratio (HNR; dB): quantifying the amount of additive noise in the voice signal.

### Statistical analysis

Data were processed using SPSS software for Windows (version 16; SPSS Chicago, III). The Kolmogorov-Smirnov statistic was used to verify the normality of data distribution. Descriptive statistics (including means and standard deviations) for the pure-tone threshold measurements (at the investigated frequencies), and for tympanometric measurements for each ear were calculated at each of the four measurement periods separately. The data regarding the hearing levels, middle ear volume, and static compliance values were evaluated using the analysis of variance (ANOVA) test. Tukey HSD post-hoc test was applied to determine at which periods the measurement changes were significant.

The speech parameters of the non-cleft and cleft groups were evaluated independently between T0 and T2 periods using paired samples *t*-test. Tests of significance were two-tailed, and the minimum level of the statistical significance was set at *p* < 0.05.

## Results

**Figure 5:**
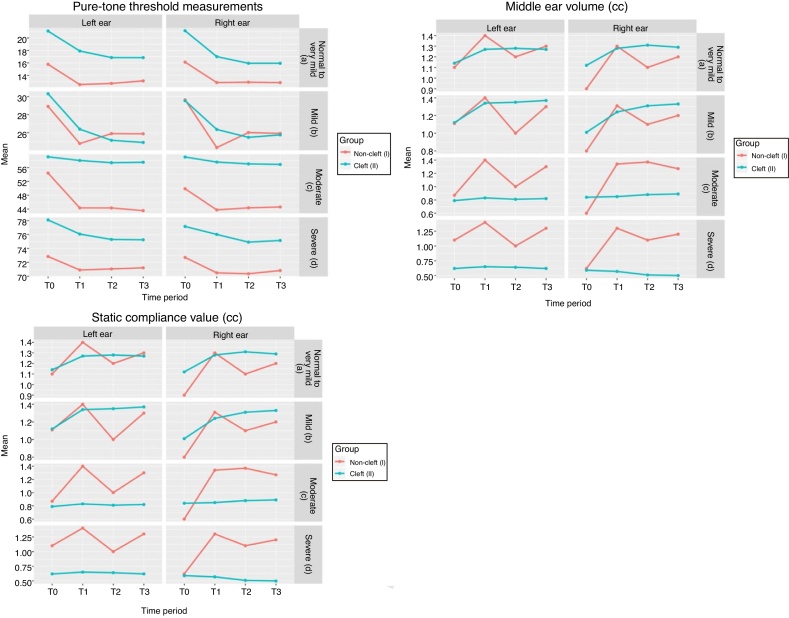
(a) Means of Pure-Tone Threshold measurements from audiograms at different time intervals in decibels among different groups. (b) Means of the middle ear volume at different time periods for individual groups. (c) Means of the static compliance values for individual groups.

The present study was proposed to evaluate the hearing ability and voice functions under the interventional effect of maxillary expansion among non-cleft and BCLP patients. Means of pure-tone threshold measurements, middle ear volume and static compliance values at different time intervals for different groups are depicted in Fig. 5a‒c respectively. Both Group Ia and Group IIa sample showed statistically significant differences for mean pure tone threshold at frequencies in both right and left ears (*p* <  0.05) (Tables 1 and S1; Fig. 5a). Significant improvements were observed in the hearing levels of both right and left ears in Group Ia during the T0–T1, T1–T2, T0–T2, and T0–T3 observation periods (Table 2). However, changes from T1–T3 and T2–T3 were not statistically significant (Table S2). Group Ib sample showed statistically significant hearing improvements in both ears from T0 to T1 observation periods, with no significant changes during other observation intervals (Tables 1, 2, S3 and S4). In Group Ic, statistically significant improvements were observed during T0–T1, T0–T2, and T0–T3 periods in the left ear (Tables 2, S5 and S6). However, in Group Id, differences between different observation periods were not statistically significant (Tables 1 and S7).

**Table 1:** Levels of Significance following ANOVA Analysis of Pure-Tone Threshold measurements from Audiogram Data.

Group classification	*p*-value		
	RE	LE	
Non-Cleft (I)	Normal to very mild (Ia)	0.0024^a^	0.0062^a^
	Mild (Ib)	0.0161^a^	0.0224^a^
	Moderate (Ic)	0.0647	0.0023^a^
	Severe (Id)	0.936	0.9638
Cleft (II)	Normal to very mild (IIa)	0.0003^a^	0.0082^a^
	Mild (IIb)	0.1962	0.0357^a^
	Moderate (IIc)	0.9212	0.9575
	Severe (IId)	0.966	0.9359

RE, Right Ear; LE, Left Ear.

a *p* < 0.05 is significant.

**Table 2:** Descriptive statistics and statistical comparisons of the significant changes in right and left ear at different time intervals within individual groups (Tukey HSD Post-hoc test).

Intragroup comparison	Mean Difference		95 % CI		*p*-value	
	RE	LE	RE	LE	RE	LE
Non-Cleft Group-Normal (Ia)						
T0 vs. T1	−3.2825	−3.2500	−5.8721 to −0.6929	−5.8837 to −0.6163	0.0081^a^	0.0103^a^
T0 vs. T2	−3.2225	−3.0700	−5.8121 to −0.6329	−5.7037 to −0.4363	0.0096^a^	0.0168^a^
T0 vs. T3	−3.2925	−2.6600	−5.8821 to −0.7029	−5.2937 to −0.0263	0.0079^a^	0.0470^a^
T1 vs. T2	0.0600	0.1800	−2.5296 to 2.6496	−2.4537 to 2.8137	0.001^a^	0.0470^a^
Cleft Group-Normal (IIa)						
T0 vs. T1	−4.1500	−3.2000	−7.4715 to −0.8285	−6.7881 to 0.3881	0.0095^a^	0.0448^a^
T0 vs. T2	−5.2200	−4.2500	−8.5415 to −1.8985	−7.8381 to −0.6619	0.0008^a^	0.0149^a^
T0 vs. T3	−5.2200	−4.2600	−8.5415 to −1.8985	−7.8481 to −0.6719	0.0008^a^	0.0146^a^
T2 vs. T3	0.0000	−0.0100	−3.3215 to 3.3215	−3.5981 to 3.5781	0.0000^a^	1.0133
Non-Cleft Group-Mild (Ib)						
T0 vs. T1	−5.2900	−4.1200	−9.5741 to −1.0059	−8.7544 to 0.5144	0.0111^a^	0.0449^a^
Cleft Group-Mild (IIb)						
T0 vs. T3	−3.8000	−5.4000	−9.4203 to 1.8203	−10.7336 to −0.0664	0.2739	0.0464^a^
Non-Cleft Group-Moderate (Ic)						
T0 vs. T1	−6.1100	−10.0700	−12.8026 to 0.5826	−17.4284 to −2.7116	0.0799	0.0061^a^
T0 vs. T2	−5.5200	−10.0800	−12.2126 to 1.1726	−17.4384 to −2.7216	0.1258	0.0060^a^
T0 vs. T3	−5.2600	−9.8500	−11.9526 to 1.4326	−17.2084 to −2.4916	0.1524	0.0072^a^

CI, Confidence Interval of difference.

a *p* < 0.05 is significant.

In cleft group patients with normal hearing levels, statistically significant hearing improvements were observed from the T0 to T1, T0 to T2 and T0 to T3 periods in both ears. (Tables 2, S8 and S9). Intragroup comparison in Group IIb revealed a significant decrease in mean pure-tone threshold (at different frequencies) measurement in the left ear only (Table S10). Comparison of the means in Group IIb revealed significant hearing improvements during the T0–T3 period in the left ear (Tables 2, S10 and S11). On the other hand, the hearing improvements were not statistically significant in both Groups IIc and IId (Tables 1, S12 and S13).

In Group Ia, right middle ear volume showed statistically significant improvement during the following observation periods: T0–T1, T1–T2 and T0–T3; while the improvements in left middle ear volume were not statistically significant (Tables 3, S14 and S15). Changes observed in the static compliance values during the widening, stabilization and retention periods were slight and insignificant in both Groups Ia and IIa (Tables 3 and S14). Tukey post hoc test showed a highly significant change (*p* = 0.000) in right middle ear volume during the T1–T3 period in Group IIa (Table S16).

**Table 3:** Levels of Significance following ANOVA Analysis of the Tympanogram Data [middle ear volume & static compliance values (cc)].

	Middle ear volume		Static Compliance value		Middle ear volume		Static Compliance value	
	RE	LE	RE	LE	RE	LE	RE	LE
	Non-Cleft – Normal to very mild (Group Ia)				Cleft – Normal to very mild (Group IIa)			
*p*-value	0.0043^a^	0.3142	0.977	0.2114	0.05^a^	0.1386	0.5415	0.8051
	Non-Cleft – Mild (Group Ib)				Cleft – Mild (Group IIb)			
*p*-value	0.0024^a^	0.0111^a^	0.9802	0.763	0.0003^a^	0.0037^a^	0.8806	0.3615
	Non-Cleft – Moderate (Group Ic)				Cleft – Moderate (Group IIc)			
*p*-value	0.0019^a^	0.1455	0.9814	0.9859	0.9304	0.9762	0.9954	0.9797
	Non- Cleft – Severe (Group Id)				Cleft – Severe (Group IId)			
*p*-value	0.1394	0.7344	0.9955	0.9966	0.9	0.9984	0.9818	0.8606

a *p* < 0.05 is significant.

Both Group Ib and Group IIb samples showed a statistically significant increase in both the right and left middle ear volumes with no significant change in static compliance values (Table S14). In Group Ib, significant change in middle ear volume was observed during the T0–T1 and T0–T3 periods in right ear and during the T1–T2 period in the left ear (Table S17). Both right and left middle ear volumes in Group IIb also showed significant increases during the T0–T1, T0–T2 and T0–T3 periods (Table S18). In Group Ic, significant mean differences were observed only for right middle ear volumes during the T0–T1, T0–T2 and T0–T3 observation periods; while the middle ear volume changes in group IIc samples were statistically insignificant (Tables 3, S14 and S19). The changes in the static compliance values were slight and statistically insignificant in Groups Ic and IIc (Tables 3 and S14). However, Groups Id and IId did not show any improvements in middle ear volumes and static compliance values (Table S14).

**Figure 6:**
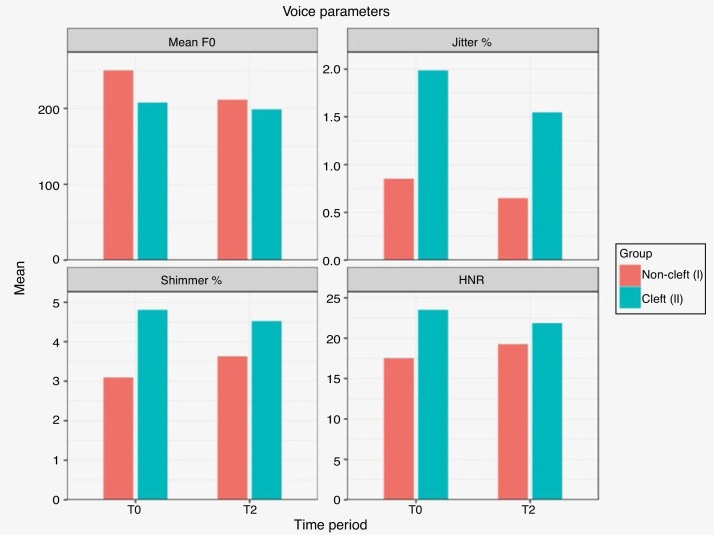
Means of different voice parameters at T0 and T2 periods.

As for the voice analysis, means of different voice parameters at T0 and T2 periods are depicted in Fig. 6. In the non-CLP group, statistically significant differences were observed only between the T0 and T2 mean F0 (significant decrease,) and jitter (%) (*p* < 0.05; Table 4). In the cleft group, no significant differences were observed for any parameter between the T0 and T2 observation periods (Table 4).

**Table 4:** Statistical comparison of voice parameters at T0 and T2 within non-cleft and cleft group (paired *t*-Test).

Classification	Non-Cleft Group					
Parameters	T0	T2	Difference	Standard error	95% Cl	*p*-value
Mean F0	249.32	210.67	−38.650	13.847	−66.6364 to −10.6636	0.0080^a^
Jitter %	0.846 ± 0.32	0.642 ± 0.29	−0.204	0.094	−0.3945 to −0.0135	0.0364^a^
Shimmer %	3.076 ± 1.43	3.612 ± 1.98	0.536	0.533	−0.5412 to 1.6132	0.3206
HNR	17.45 ± 3.7	19.178 ± 4.31	1.728	1.240	−0.7772 to 4.2332	0.1710
	Cleft Group					
Mean F0	206.67 ± 51.6	197.87 ± 45.6	−8.800	13.772	−36.4911 to 18.8911	0.5259
Jitter %	1.98 ± 0.52	1.54 ± 3.2	−0.440	0.648	−1.7437 to 0.8637	0.5007
Shimmer %	4.789 ± 1.96	4.507 ± 0.98	−0.282	0.438	1.1632 to 0.5992	0.5230
HNR	23.43 ± 5.76	21.78 ± 4.97	−1.650	1.522	−4.7093 to 1.4093	0.2836

F0, Fundamental frequency; HNR, Harmonic to Noise ratio; df, degrees of freedom.

a *p* <  0.05 (significant).

## Discussion

Correction of transverse maxillary deficiency by arch expansion is considered to be one of the foremost important steps in the rehabilitation of any orthodontic and dentofacial deformity, both in non-cleft and cleft lip palate affected constrictions. Owing to combination of favourable dentofacial and craniofacial treatment effects,^27^ RME has proven to be a biologically and biomechanically sound treatment modalities for the correction of posterior crossbites, dental crowding, nasal stenosis, abnormal breathing pattern and CHL in growing children presenting with transverse maxillary constriction.^17^ The mechanism linking maxillary expansion and hearing improvement may be explained by the favourable soft tissue changes accompanying correction of the palatal anatomy by RME. Studies have shown that RME influences the muscular function of tubal ostia, wherein stretching of the elevator and Tensor Veli Palatine muscles to open the pharyngeal orifice of the ET, thus allowing air to enter or leave the middle ear through the tube. This ensures balanced pressures on either side of the tympanic membrane, free vibration of the ossicular chain, thus allowing for the correct function of the tympanic membrane and the auditory system.^6^, ^12^, ^28^, ^29^

In the present study, we used bonded Hyrax expander with occlusal acrylic coverage because of its advantages of allowing greater skeletal expansion, more parallel movement of the anchor teeth and the two maxillary halves, and long-term stability as compared to the conventional expanders.^5^ Necessary steps were taken to ensure the adequacy of blinding at the operator level to eliminate the influence of unknown confounding factors and obtain unbiased results. All the subjective and objective assessments (hearing records and voice function) were performed under blinded conditions to help eliminate the influence of cognitive bias on the results in the assessments.

As for the methods of hearing evaluation, a pure-tone audiogram is considered a vital tool for the subjective assessment of hearing levels and for measuring the level of recognition of pure-tone sounds at different frequencies.^17^ Comparative measurements of air- and bone conduction thresholds across all the hearing frequencies using a pure-tone audiogram helps distinguish a conductive hearing loss from a sensorineural type of hearing loss.^30^

Objective audiometric assessment using tympanometry provides valuable quantitative information about the compliance or mobility of the tympanic membrane, the presence of fluid in the middle ear, mobility and pressure within the middle ear system, ET dysfunctions and ear canal volume. Measurement of the static compliance of the tympanic membrane and of the volume of the tympanic cavity by the use of tympanograms proves beneficial in determining the changes in stiffness of the tympanic membrane, reduction of the middle ear effusion, and volumetric changes in the middle ear cavity.^31^, ^32^, ^33^ By virtue of its non-invasiveness and sensitiveness with multiple diagnostic advantages, tympanometric records were also included in our study for investigating the changes in hearing.

The results of the present study highlight that all non-cleft and cleft subjects with normal hearing levels and with mild conductive hearing loss, selected for palatal expansion showed significant improvements in hearing levels during the T0–T1, T1–T2, T0–T2, and T0–T3 observation periods. However, improvements in hearing after maxillary expansion did not show a further significant increase but were retained during the T1–T2 and T2–T3 observation periods. Despite general consensus regarding the positive effects of RME on hearing levels, conflicting reports exist in literature regarding the maintenance of hearing improvements over a period of time; with few authors^12^, ^14^, ^34^ demonstrating retention of the hearing improvements only (without any significant increase), while others^15^, ^35^ reporting some relapse of the results obtained after expansion during the retention period. These variations in the short- and long-term hearing improvements could be attributed to variability in the growth status of the selected sample, the difference in the appliances used and the inevitable relapse tendency for Hyrax appliances.

However, the cleft group and the non-cleft group patients with moderate to severe hearing loss did not show statistically significant hearing improvement during the observation period from T0–T1 to T2–T3, except for the significant improvements in left ear during T0–T1, T0–T2 and T0–T3 periods in non-cleft group patients exhibiting moderate hearing loss.

Regarding the results of the tympanometric evaluation, middle ear volumes of both the non-cleft and cleft group patients with normal hearing levels and mild hearing loss showed considerable improvement during all the observation periods. Similar findings were also reported in the study of Kilic et al.^5^ and Villano et al.,^29^ involving non-cleft patients with bilateral posterior crossbites. However, in non-cleft group with a moderate hearing loss, only right middle ear volumes during the T0–T1, T0–T2 and T0–T3 observation periods showed significant improvements. The middle ear volume changes in non-cleft and cleft group samples with severe hearing loss were statistically insignificant. Slight but insignificant increases were observed in the static compliance values in both CLP and non-CLP group patients with normal hearing levels and moderate hearing losses. However, no improvements were observed in the middle ear volumes and static compliance values in all patients with severe hearing loss. This may be attributed to the increased role of anatomical anomalies of the tensor veli palatini muscle and morphological variations in the surrounding structures influencing ET dysfunction in such patients.^36^

These findings are in accordance with those of Handzik-Cuk et al.^37^ who also reported that hearing level in untreated patients with BCLP showed improvement only in groups with hearing levels of 21–40 dB, while those with hearing levels above 40 dB i.e. moderate and severe hearing loss showed no significant improvement with age. As the volume of the cleft and local postoperative tension are greater in BCLP than in Unilateral Cleft Lip and Palate (UCLP), the patients with BCLP exhibit a much more impressive compensatory mechanism of pharyngeal wall movements on deglutition and phonation (Passavant’s and circular fold) compared to the other cleft types, which contributes to mechanical obstruction of the pharyngeal orifice of the ET. Additionally, development of the middle face and ET is further slowed in these patients due to disturbed aeration of the paranasal sinuses, along with local postoperative tension.^37^

In a three-dimensional finite element analysis of ET function under normal and pathological conditions, Sheer et al.^38^ concluded that changes in cartilage and periluminal mucosal tissue rather than changes in tensor veli palatini muscle forces might be of greater importance in proper ET function in young children with cleft anomalies. Apart from dysfunctions of the tensor veli palatini muscle in patients with cleft palate, alterations in skull base and pharyngeal anatomy are other factors leading to malfunction of the ET.^39^ However, the extent of the contribution of each factor to ET dysfunction and otitis media with effusion in cleft palate patients remains unclear.

Tunçbilek et al.^40^ reported that the final hearing status of patients with cleft palate is governed by a combination of surgical correction, developmental factors, and treatment of middle ear disease. Although mechanisms are still to be uncovered, it has been theorized that preservation of the tensor veli palatini muscle integrity during cleft palate repair seems to improve the long-term otologic outcome of cleft palate patients. However, the results of the studies assessing the role of the tensor veli palatini muscle in ET opening in non-cleft patients should be extrapolated with caution in bilateral cleft palate patients due to variations in anatomy, severe malposition and tissue deficiency of the orofacial region encountered in cleft patients.^36^

Fagundes et al.^17^ observed that most of the non-randomized studies reporting improvements in hearing levels varying from approximately 2–19 dB after RME, presented low to moderate biases due to considerable variation in sample characteristics, treatment features, methods of hearing level evaluation and follow-up period. Only a few studies classified the level of hearing impairment before RME intervention but showed no differences in the final hearing assessments.^17^ The present study also evaluated laryngeal voice by objective acoustic analysis using PRAAT software, which offers the advantages of being easy to use, rapid, economical, reliable, reproducible and non-invasive in pediatric patients.^18^, ^41^ Since the RME appliance causes alterations in the speech by affecting the tongue posture and palatal volume,^42^ voice recordings were performed at T2 after removal of the appliance.

Measurements of vocal extension profile, fundamental frequency, perturbation index (jitter and shimmer) and harmonics-to-noise ratio are the most important vocal acoustic parameters having clinical implications.^43^ Fundamental frequency, usually representing the length of the vocal folds, is determined physiologically by the number of cycles that the vocal folds make in a second. The variations that occur in the fundamental frequency are represented by jitter and shimmer parameters. While jitter indicates the variability or perturbation of fundamental frequency, shimmer refers to the same perturbation in relation to the amplitude of the sound wave, or intensity of vocal emission.^44^ Lack of control of vocal fold vibration, as observed in pathological voices usually affects jitter percentage.^45^ Lewis et al.^46^ reported that jitter and shimmer values are associated with resistance of the laryngeal airway and incomplete velopharyngeal closure in cleft lip palate patients.

For examining voice quality, Harmonic-to-Noise Ratios (HNR) were obtained for vowels in the conversation, conversation-repetition, and the first and second sentence repetition samples using PRAAT software. Normalization of the HNR values (nHNR) was done using the duration of the measured vowel portions. All the voice recordings were blocked and randomized for assessment to eliminate the influence of cognitive bias on the results in the assessments.

Due to lowered and backward positioning of the tongue during phonation of the /a/ vocal, the closure and constriction in the center of the vocal tract do not occur as in the other voices. Additionally, since the/a/sound also constitutes the phonological core of many syllables,^47^ we assessed parameters of the vowel/a/, in evaluating the voice. Moreover, based on the recommendations of Moura et al.,^18^ acoustic analysis of voice in the present study relied on a sustained vowel task to avoid any possibility of interference with the control of speech prosody and articulation.

Regarding the effects of RME on speech, the results of the present study are difficult to compare to previous investigations since objective studies assessing the influence of RME on vocal function in subjects with bilateral cleft palate are relatively non-existent. Moreover, heterogeneity in methodology, mixed patient groups and non-unified surgical techniques make comparison of speech results of different studies difficult. Flynn et al.^48^ reported higher prevalence of abnormal middle ear status and decreased hearing abilities among individuals treated for CLP to be a major factor potentially affecting speech in such patients. Van Lierde et al.^49^ postulated that children with cleft palate have an increased predisposition to voice disorders because of the more intensive vocal tract activities compared with noncleft subjects. Decreased vocal quality in these patients may be caused by a direct laryngeal pathology frequently manifesting as bilateral vocal nodules or muscle tension pattern type I, or may be a compensatory indirect response to a velopharyngeal disorder.

In the current study, after appliance removal, the cleft group showed a statistically insignificant decrease in mean F0 frequency. Contrary to the results of Gonzalez et al.^11^ who reported an increase in F0 and higher shimmer perturbation in repaired unilateral cleft lip and palate cases, we observed no statistically significant changes in the measurements of F0, jitter or shimmer percentages, and HNR after expansion in BCLP patients. However, in the non-cleft group, a significant decrease in F0 and jitter percentage was observed. In accordance with the findings of Bilgic et al.,^19^ significant changes in F0 and jitter percentage in the non-cleft group in our study indicate that the application of RME affects voice quality in non-cleft patients. Thus, the possible effects on voice changes need to be explained to the patients and parents before initiation of RME therapy in non-cleft patients.

Macari et al.^50^ also demonstrated the significant influence of RME on voice function in pediatric patients. However, Yurttadur et al.^20^ detected no significant changes in F0, jitter or shimmer percentages and Noise-to-harmonic ratio parameters, thus suggesting no changes in the voice quality or resonance after RME therapy. The difference in the outcome could be attributed to a wider age interval (from 12 to 17 years) of the subjects chosen in that study.

As for the limitations of the study, a non-expanded control group with posterior cross-bite was not constituted due to ethical reasons for denying the benefits of timely correction of functional crossbite. Furthermore, the results of the present study should be extrapolated with caution and a greater number of randomized trials can be planned and conducted in different populations with larger sample size and involving long follow-up periods to further validate the findings. Moreover future studies can take benefit of advanced diagnostic AID such as video-otoscopy for instrumental observation of the external auditory tube and the tympanic membrane.

## Conclusions

This study showed that the correction of the palatal anatomy by RME therapy had a positive and statistically significant effect on improvements in hearing and function of the middle ear in both non-cleft and bilateral cleft lip palate patients with normal hearing levels and with mild conductive hearing loss. Similarly, RME significantly influenced voice quality in non-cleft patients, with no significant effect in bilateral cleft lip palate patients.

## Conflicts of interest

The authors declare no conflicts of interest.
