# Supplementary material for: Effects of maxillary expansion on hearing and voice function in non-cleft lip palate and cleft lip palate patients with transverse maxillary deficiency: a multicentric randomized controlled trial
Source: Braz J Otorhinolaryngol. 2019 Nov 2;87(3):315–25. doi: 10.1016/j.bjorl.2019.09.010 (PMC9422610; doi:10.1016/j.bjorl.2019.09.010)
Supplement: Supplementary file 1 [file mmc1.docx]

**BJORL-D-19-00274_SUPPLEMENTARY TABLES**

| **Classification** | **Non-Cleft Group-Normal to very mild** | | | | | | | | | |
| --- | --- | --- | --- | --- | --- | --- | --- | --- | --- | --- |
| **Frequency (Hz)** | **250** | | **500** | | **1000** | | **2000** | |  |  |
|  | **RE** | **LE** | **RE** | **LE** | **RE** | **LE** | **RE** | **LE** | **Mean RE** | **Mean LE** |
| **To** | 22.23+  3.2 | 22+  2.9 | 17.1+  2.3 | 16.9+  3.1 | 14+  2.9 | 13.33+  1.5 | 11.12+  1.8 | 10.89+  2.2 | 16.1325  +  2.55 | 15.78 +  2.425 |
| **T1** | 18+  4.2 | 19+  3.5 | 13.13+  1.9 | 12.85+  2.7 | 11.15+  2.1 | 10.16+  2.1 | 9.12 +  3 | 8.13+  1.8 | 12.85 +  2.8 | 12.53+  2.52 |
| **T2** | 17.98+  1.2 | 20+  1.2 | 12.9+  1.2 | 12.76+  2.2 | 12.08+  1.9 | 10.04+  3.1 | 8.7+  1.8 | 8.05+  1.9 | 12.91+  1.52 | 12.71 +2.1 |
| **T3** | 17.74+  0.5 | 20+  0.5 | 12.78+  2.1 | 12.96+  3.2 | 12.06+  2.5 | 11.11+  2.8 | 8.81+  2.8 | 8.43+  2.1 | 12.84+  1.97 | 13.12+  2.15 |
| ***P* value** | | | | | | | | | **0.0024*** | **0.0062*** |
| ***F* value** | | | | | | | | | 5.7157 | 4.7668 |

**Table S1**. Means and standard deviation of ­measurements from audiograms at different speech. frequencies and time intervals in decibels among group Ia (ANOVA test)

T0 – before RME; T1 – at the end of RME; T2 – 3 months after RME; and T3 – at the end of 6 months; RE, right ear; LE, left ear; **P*< 0.05 (significant)

**Table S2.** Results of Tukey HSD posthoc Test explaining the significances in variance analyses for group Ia.

| **Intragroup Comparison** | **Mean Difference** | | | **95 % CI** | | ***P* value** | |
| --- | --- | --- | --- | --- | --- | --- | --- |
|  | **RE** | | **LE** | **RE** | **LE** | **RE** | **LE** |
| **To vs T1** | -3.2825 | -3.2500 | | -5.8721 to -0.6929 | -5.8837 to -0.6163 | **0.0081*** | **0.0103*** |
| **To vs T2** | -3.2225 | -3.0700 | | -5.8121 to -0.6329 | -5.7037 to -0.4363 | **.0096*** | **0.0168*** |
| **To vs T3** | -3.2925 | -2.6600 | | -5.8821 to -0.7029 | -5.2937 to -0.0263 | **0.0079*** | **0.0470*** |
| **T1 vs T2** | 0.0600 | 0.1800 | | -2.5296 to 2.6496 | -2.4537 to 2.8137 | **0.001*** | **0.0470*** |
| **T1 vs T3** | 0.0100 | 0.5900 | | -2.5996 to 2.5796 | -2.0437 to 3.2237 | 1.4295 | 0.9313 |
| **T2 vs T3** | 0.0700 | 0.4100 | | -2.6596 to 2.5196 | -2.2237 to 3.0437 | 0.9998 | 0.9752 |

CI, confidence interval of difference

| **Classification** | **Non-Cleft Group-Mild** | | | | | | | | | |
| --- | --- | --- | --- | --- | --- | --- | --- | --- | --- | --- |
| **Frequency (Hz)** | **250** | | **500** | | **1000** | | **2000** | |  |  |
|  | **RE** | **LE** | **RE** | **LE** | **RE** | **LE** | **RE** | **LE** | **Mean RE** | **Mean LE** |
| **To** | 32+  3.2 | 31.56+  3.2 | 30.32±  2.7 | 29.01±  5.3 | 28.76+  4.3 | 27.18+  3.1 | 27.48+  2.9 | 27.93+  3.7 | 29.64+  3.27 | 28.92+  3.82 |
| **T1** | 24.94+  1.8 | 25+  1.8 | 23.34±  3.4 | 24.75±  4.9 | 24.01+  3.9 | 25.23+  3.9 | 25.12+  3.1 | 24.25+  3.3 | 24.35+  3.1 | 24.80+  3.47 |
| **T2** | 28+  2.0 | 28+  2.0 | 25.32+  3.5 | 24.78+  3.2 | 25.67+  2.8 | 25.86+  2.9 | 25.11+  3.4 | 24.98+  2.9 | 26.02+  2.92 | 25.90+  2.75 |
| **T3** | 27+  3.4 | 27+  3.4 | 25.67+  2.3 | 25.5±  4.6 | 25.92+  3.2 | 26.13+  2.7 | 25.09+  4.1 | 24.91+  3.1 | 25.92+  3.25 | 25.88+  3.45 |
| ***P* value** | | | | | | | | | **0.0161*** | **0.0224*** |
| ***F* value** | | | | | | | | | 4.0747 | 2.1816 |

**Table S3.** Means and standard deviation of measurements from audiograms at different speech frequencies and time intervals in decibels among group Ib (ANOVA test).

**Table S4**. Results of Tukey HSD posthoc Test explaining the significances in variance analyses for group Ib.

| **Intragroup comparison** | **Mean Difference** | | | **95 % CI** | | ***P* value** | |
| --- | --- | --- | --- | --- | --- | --- | --- |
|  | **RE** | | **LE** | **RE** | **LE** | **RE** | **LE** |
| **To vs T1** | -5.2900 | -4.1200 | | -9.5741 to -1.0059 | -8.7544 to 0.5144 | **0.0111*** | **0.0449*** |
| **To vs T2** | -3.6200 | -3.0200 | | -7.9041 to 0.6641 | -7.6544 to 1.6144 | 0.1206 | 0.3041 |
| **To vs T3** | -3. 7200 | -3.0400 | | -8.0041 to 0.5641 | -7.6744 to 1.5944 | 0.1063 | 0.2987 |
| **T1 vs T2** | 1.6700 | 1.1000 | | -2.5296 to 2.6496 | -3.5344 to 5.7344 | 0.7136 | 0.9153 |
| **T1 vs T3** | 1.5700 | 1.0800 | | -2.5996 to 2.5796 | -3.5544 to 5.7144 | 0.7503 | 0.9194 |
| **T2 vs T3** | -0.1000 | -0.0200 | | -2.6596 to 2.5196 | -4.6544 to 4.6144 | 0.9999 | 1.2872 |

**Table S5**. Means and standard deviation of measurements from audiograms at different speech frequencies and time intervals in decibels among group Ic (ANOVA test).

| **Classification** | **Non-Cleft Group-Moderate** | | | | | | | | | |
| --- | --- | --- | --- | --- | --- | --- | --- | --- | --- | --- |
| **Frequency (Hz)** | **250** | | **500** | | **1000** | | **2000** | |  |  |
|  | **RE** | **LE** | **RE** | **LE** | **RE** | **LE** | **RE** | **LE** | **Mean RE** | **Mean LE** |
| **To** | 59+  3.2 | 59+  3.2 | 52.15+  4.3 | 51.74+  4.7 | 45.16+  5.1 | 46.25+  5.6 | 43.17+  3.6 | 43.18+  4.1 | 49.87  4.05 | 54.40  4.4 |
| **T1** | 54+  1.9 | 54+  1.9 | 44.78+  3.9 | 45.67+  5.1 | 39.18+  4.3 | 39.54+ 5.5 | 37.08+  3.9 | 38.14+  4.7 | 43.76  3.5 | 44.33  4.3 |
| **T2** | 55+  2.4 | 55+  2.4 | 45.13+  3.2 | 45.21+  4.3 | 40.11+  4.6 | 38.96+  4.1 | 37.17+  4.1 | 38.11+  3.8 | 44.35  3.57 | 44.32  3.65 |
| **T3** | 54+  .7 | 54+  1.7 | 46.11+  4.1 | 45.01+  4.6 | 40.16+  5.1 | 37.17+  4.9 | 38.18+  4.7 | 38.05+  4.3 | 44.61  3.65 | 43.55  3.87 |
| ***P* value** | | | | | | | | | 0.0647 | **0.0023*** |
| ***F* value** | | | | | | | | | 2.9425 | 7.5621 |

**Table S6.** Results of Tukey HSD posthoc Test explaining the significances in variance analyses for group Ic.

| **Intragroup Comparison** | **Mean Difference** | | | **95 % CI** | | ***P* value** | |
| --- | --- | --- | --- | --- | --- | --- | --- |
|  | **RE** | | **LE** | **RE** | **LE** | **RE** | **LE** |
| **To vs T1** | -6.1100 | -10.0700 | | -12.8026 to 0.5826 | -17.4284 to -2.7116 | 0.0799 | **0.0061*** |
| **To vs T2** | -5.5200 | -10.0800 | | -12.2126 to 1.1726 | -17.4384 to -2.7216 | 0.1258 | **0.0060*** |
| **To vs T3** | -5.2600 | -9.8500 | | -11.9526 to 1.4326 | -17.2084 to -2.4916 | 0.1524 | **0.0072*** |
| **T1 vs T2** | 0.5900 | -0.0100 | | -6.1026 to 7.2826 | -7.3684 to 7.3484 | 0.9941 | 2.3771 |
| **T1 vs T3** | 0.8500 | 0.2200 | | -5.8426 to 7.5426 | -7.1384 to 7.5784 | 0.9830 | 0.9998 |
| **T2 vs T3** | 0.2600 | 0.2300 | | -6.4326 to 6.9526 | -7.1284 to 7.5884 | 0.9995 | 0.9997 |

**Table S7.** Means and standard deviation of measurements from audiograms at different speech frequencies and time intervals in decibels among group Id (ANOVA test).

| **Classification** | **Non-Cleft Group-Severe** | | | | | | | | | |
| --- | --- | --- | --- | --- | --- | --- | --- | --- | --- | --- |
| **Frequency (Hz)** | **250** | | **500** | | **1000** | | **2000** | |  |  |
|  | **RE** | **LE** | **RE** | **LE** | **RE** | **LE** | **RE** | **LE** | **Mean RE** | **Mean LE** |
| **To** | 75+  2.2 | 75+  2.2 | 72.97+  3.4 | 71.56+  3.9 | 71.78+  4.7 | 72.28+  5.1 | 71.09+  5.3 | 72.55+  4.9 | 72.71+  3.9 | 72.84+  4.02 |
| **T1** | 73+  3.8 | 73+  3.8 | 69.15+  4.1 | 70.07+  4.1 | 71.11+  4.5 | 69.94+  4.7 | 68.75+  5.3 | 70.64+  5.4 | 70.50+  4.42 | 70.91+  4.5 |
| **T2** | 73+  2.9 | 73+  2.9 | 69.26+  4.3 | 70.12+  3.8 | 70.86+  5.1 | 70.51+  5.6 | 68.37+  5.9 | 70.61+  5.1 | 70.37+  4.55 | 71.06+  4.35 |
| **T3** | 74+  2.3 | 74+  2.3 | 69.12+  3.6 | 70.67+  4.1 | 69.97+  5.3 | 70.04+  5.4 | 68.61+  5 | 70.18+  5.7 | 70.83+  4.05 | 71.22+  4.37 |
| ***P* value** | | | | | | | | | 0.9360 | 0.9638 |
| ***F* value** | | | | | | | | | 0.1320 | 0.0865 |

**Table S8**. Means and standard deviation of measurements from audiograms at different speech frequencies and time intervals in decibels among group IIa (ANOVA test).

| **Classification** | **Cleft Group-Normal to very mild** | | | | | | | | | |
| --- | --- | --- | --- | --- | --- | --- | --- | --- | --- | --- |
| **Frequency (Hz)** | **250** | | **500** | | **1000** | | **2000** | |  |  |
|  | **RE** | **LE** | **RE** | **LE** | **RE** | **LE** | **RE** | **LE** | **Mean RE** | **Mean LE** |
| **To** | 24+  4.1 | 25+  3.2 | 22.12+  2.3 | 21.98+  3.1 | 19.67+  2.9 | 19.78+  2.9 | 18.88+  3.1 | 17.68+  2.9 | 21.16+  3.1 | 21.11+  3.02 |
| **T1** | 19+  0.98 | 23+  1.3 | 18.32+  3.1 | 18.38+  3.7 | 15.26+  3.2 | 16.12+  2.1 | 15.47+  3.3 | 14.14+  3.4 | 17.01+  2.64 | 17.91+  2.62 |
| **T2** | 20+  1.2 | 21+  2.8 | 16.23+  3.3 | 16.1+  2.9 | 14.87+  3.4 | 16.08+  3.1 | 14.58+  2.9 | 14.28+  3.1 | 15.94+  2.7 | 16.86+  2.97 |
| **T3** | 20+  1.2 | 22+  3.6 | 16.43+  2.7 | 15.98+  3.0 | 13.29+  3.1 | 15.97+  2.6 | 14.05+  3.4 | 13.47+  3.9 | 15.94+  2.6 | 16.85+  3.27 |
| ***P* value** | | | | | | | | | **0.0003*** | **0.0082*** |
| ***­­ F* value** | | | | | | | | | 8.1097 | 4.5707 |

**Table S9**. Results of Tukey HSD posthoc test explaining the significances in variance analyses for group IIa.

| **Intragroup comparison** | **Mean difference** | | **95 % CI** | | ***P* value** | |
| --- | --- | --- | --- | --- | --- | --- |
|  | **RE** | **LE** | **RE** | **LE** | **RE** | **LE** |
| **To vs T1** | -4.1500 | -3.2000 | -7.4715 to -0.8285 | -6.7881 to 0.3881 | **0.0095*** | **0.0448*** |
| **To vs T2** | -5.2200 | -4.2500 | -8.5415 to -1.8985, | -7.8381 to -0.6619 | **0.0008*** | **0.0149*** |
| **To vs T3** | -5.2200 | -4.2600 | -8.5415 to -1.8985, | -7.8481 to -0.6719 | **0.0008*** | **0.0146*** |
| **T1 vs T2** | -1.0700 | -1.0500 | -4.3915 to 2.2515 | -4.6381 to 2.5381 | 0.8214 | 0.8593 |
| **T1 vs T3** | -1.0700 | -0.1600 | -4.3915 to 2.2515 | -4.6481 to 2.5281 | 0.8214 | 0.8559 |
| **T2 vs T3** | 0.0000 | -0.0100 | -3.3215 to 3.3215 | -3.5981 to 3.5781 | **0.0000*** | 1.0133 |

**Table S10**. Means and standard deviation of measurements from audiograms at different speech frequencies and time intervals in decibels among group IIb (ANOVA test).

| **Classification** | **Cleft group-Mild** | | | | | | | | | |
| --- | --- | --- | --- | --- | --- | --- | --- | --- | --- | --- |
| **Frequency (Hz)** | **250** | | **500** | | **1000** | | **2000** | |  |  |
|  | **RE** | **LE** | **RE** | **LE** | **RE** | **LE** | **RE** | **LE** | **Mean RE** | **Mean LE** |
| **To** | 32+  4.3 | 34+  5.1 | 30.42+  4.2 | 31.87+  3.2 | 28.78+  3.1 | 27.91+  2.9 | 27.05+  3.1 | 27.51+  3.9 | 29.56+  3.67 | 30.32+  3.77 |
| **T1** | 28+  3.9 | 29+  2.7 | 27.18+  4.8 | 27.96+  4.2 | 26.12+  3.6 | 24.95+  4.1 | 24.17+  3.9 | 23.65+  4.1 | 26.36+  4.05 | 26.39+  3.77 |
| **T2** | 26+  4.4 | 28+  4.1 | 26.07+  3.7 | 26.13+  3.7 | 26.05+  3.7 | 23.87+  4.3 | 23.87+  4.2 | 22.65+  3.4 | 25.49+  4.15 | 25.16+  3.85 |
| **T3** | 27+  5.3 | 27+  3.9 | 25.94+  4.1 | 26.2+  4.3 | 27.01+  4.1 | 23.78+  3.8 | 23.1+  4.7 | 22.71+  4.9 | 25.76+  4.55 | 24.92+  4.22 |
| ***P* value** | | | | | | | | | 0.1962 | **0.0357*** |
| ***F* value** | | | | | | | | | 1.6691 | 3.2740 |

**Table S11**. Results of Tukey HSD posthoc test explaining the significances in variance analyses for group IIb.

| **Intragroup comparison** | **Mean difference** | | | **95 % CI** | | ***P* value** | |
| --- | --- | --- | --- | --- | --- | --- | --- |
|  | **RE** | | **LE** | **RE** | **LE** | **RE** | **LE** |
| **To vs T1** | -3.2000 | -3.9300 | | -8.8203 to 2.4203 | -9.2636 to 1.4036 | 0.4200 | 0.2078 |
| **To vs T2** | -4.0700 | -5.1600 | | -9.6903 to 1.5503 | -10.4936 to 0.1736 | 0.2205 | 0.0607 |
| **To vs T3** | -3.8000 | -5.4000 | | -9.4203 to 1.8203 | -10.7336 to -0.0664 | 0.2739 | **0.0464*** |
| **T1 vs T2** | -0.8700 | - 1.2300 | | -6.4903 to 4.7503, | -6.5636 to 4.1036 | 0.9741 | 0.9216 |
| **T1 vs T3** | -0.6000 | - 1.4700 | | -6.2203 to 5.0203 | - 6.8036 to 3.8636 | 0.9912 | 0.8748 |
| **T2 vs T3** | 0.2700 | - 0.2400 | | -5.3503 to 5.8903 | - 5.5736 to 5.0936 | 0.9992 | 0.9993 |

**Table S12**. Means and standard deviation of measurements from audiograms at different speech frequencies and time intervals in decibels among group IIc (ANOVA test).

| **Classification** | **Cleft group-Moderate** | | | | | | | | | |
| --- | --- | --- | --- | --- | --- | --- | --- | --- | --- | --- |
| **Frequency (Hz)** | **250** | | **500** | | **1000** | | **2000** | |  |  |
|  | **RE** | **LE** | **RE** | **LE** | **RE** | **LE** | **RE** | **LE** | **Mean RE** | **Mean LE** |
| **To** | 61+  4.4 | 61+4  .4 | 59.65+  7.6 | 59.54+  6.1 | 58.76+  7.1 | 58.66+  7.2 | 56.74+  5.9 | 57.17+  6.3 | 59.03+  6.25 | 59.09+  5.9 |
| **T1** | 60+  4.8 | 59+  5.6 | 58.15+  6.7 | 58.49+  6.9 | 57.96+  6.5 | 57.69+  6.8 | 54.17+  6.4 | 56.97+  6.6 | 57.57+  6.1 | 58.03+  6.47 |
| **T2** | 58+  3.6 | 57.9+  3.5 | 58.07+  7.3 | 58.66+  6.5 | 58.12+  7.3 | 57.12+  6.4 | 53.98+  6.9 | 55.87+  6.6 | 57.04+  6.27 | 57.38+  5.75 |
| **T3** | 59+  3.8 | 59.1+  4.8 | 58.66+  4.8 | 57.9+  5.4 | 57.1+  6.1 | 56.96+  7.1 | 52.79+  6.1 | 56.1+  7.1 | 56.88+  5.2 | 57.51+  5.62 |
| ***P* value** | | | | | | | | | 0.9212 | 0.9575 |
| ***F* value** | | | | | | | | | 0.1612 | 0.1027 |

**Table S13.** Means and standard deviation of measurements from audiograms at different speech frequencies and time intervals in decibels among group IId (ANOVA test).

| **Classification** | **Cleft group-Severe** | | | | | | | | | |
| --- | --- | --- | --- | --- | --- | --- | --- | --- | --- | --- |
| **Frequency (Hz)** | **250** | | **500** | | **1000** | | **2000** | |  |  |
|  | **RE** | **LE** | **RE** | **LE** | **RE** | **LE** | **RE** | **LE** | **Mean RE** | **Mean LE** |
| **To** | 81+  4.2 | 83+  5.2 | 78.67+  5.7 | 78.76+  6.7 | 75.78+  5.8 | 76.87+  6.7 | 73.17+  6.4 | 73.76+  6.8 | 77.15+  5.52 | 78.09+  6.35 |
| **T1** | 79+  5.8 | 81+  4.8 | 76.39+  6.1 | 77.17+  6.1 | 73.32+  6.2 | 74.96+  6.5 | 72.91+  6.1 | 71.12+  6.6 | 76.01+  6.12 | 76.06+  6 |
| **T2** | 78+  5.2 | 80+  6.5 | 77.1+  7.4 | 77.0+  6.8 | 72.49+  5.9 | 74.1+  5.8 | 72.02+  6.9 | 70.07+  6.1 | 74.90+  6.35 | 75.29+  6.3 |
| **T3** | 79+  6.3 | 79+  6.9 | 77.01+  5.9 | 78.38+  6.1 | 71.96+  7.1 | 73.17+  6.8 | 72.62+  6.1 | 70.43+  5.9 | 75.14+  6.1 | 75.24+  6.42 |
| ***P* value** | | | | | | | | | 0.9660 | 0.9359 |
| ***F* value** | | | | | | | | | 0.0856 | 0.1358 |

**Table S14.** Means and standard deviations of the middle ear volume & static compliance values (cc) for individual groups (ANOVA test).

| **Time Periods** | **Middle ear volume** | | **Static Compliance value** | | **Middle ear volume** | | **Static Compliance value** | |
| --- | --- | --- | --- | --- | --- | --- | --- | --- |
|  | **RE** | **LE** | **RE** | **LE** | **RE** | **LE** | **RE** | **LE** |
|  | **Non- Cleft Group-Normal to very mild (group Ia)** | | | | **Cleft Group-Normal to very mild (group IIa)** | | | |
| **T0** | 0.9±0.2 | 1.1±0.2 | 0.75±0.22 | 0.68±0.15 | 1.12±0.21 | 1.14±0.13 | 0.67±0.17 | 0.69±0.16 |
| **T1** | 1.3±0.4 | 1.4±0.6 | 0.74±0.43 | 0.67±0.17 | 1.28±0.12 | 1.27±0.15 | 0.74±0.12 | 0.76±0.21 |
| **T2** | 1.1±0.2 | 1.2±0.2 | 0.79±0.34 | 0.72±0.11 | 1.31±0.18 | 1.28±0.11 | 0.75±0.14 | 0.74±0.11 |
| **T3** | 1.2±0.1 | 1.3±0.4 | 0.78±0.15 | 0.79±0.14 | 1.29±0.14 | 1.27±0.20 | 0.74±0.11 | 0.75±0.19 |
| ***P*-value** | **0.0043*** | 0.3142 | 0.9770 | 0.2114 | **0.05*** | 0.1386 | 0.5415 | 0.8051 |
|  | **Non-Cleft Group -Mild (group Ib)** | | | | **Cleft Group – Mild (group IIb)** | | | |
| **T0** | 0.8±0.2 | 1.11±0.11 | 0.75±0.17 | 0.69±0.18 | 1.01±0.10 | 1.12±0.15 | 0.67±0.19 | 0.66±0.10 |
| **T1** | 1.31±0.4 | 1.4± 0.15 | 0.77±0.12 | 0.74±0.13 | 1.24±0.16 | 1.34±0.12 | 0.69±0.13 | 0.71±0.11 |
| **T2** | 1.1±0.2 | 1.0± 0.2 | 0.74±0.16 | 0.76±0.11 | 1.31±0.17 | 1.35±0.17 | 0.71±0.17 | 0.72±0.13 |
| **T3** | 1.2±0.1 | 1.3± 0.4 | 0.76±0.14 | 0.77±0.15 | 1.33±0.12 | 1.37±0.11 | 0.73±0.12 | 0.77±0.14 |
| ***P-*value** | **0.0024*** | **0.0111*** | 0.9802 | 0.763 | **0.0003*** | **0.0037*** | 0.8806 | 0.3615 |
|  | **Non- Cleft Group-Moderate (group Ic)** | | | | **Cleft Group-Moderate (group IIc)** | | | |
| **T0** | 0.6±0.2 | 0.87±0.2 | 0.42±0.10 | 0.38±0.19 | 0.84±0.15 | 0.79±0.17 | 0.54±0.10 | 0.58±0.15 |
| **T1** | 1.34±0.5 | 1.4±0.6 | 0.45±0.14 | 0.39±0.10 | 0.85±0.11 | 0.83±0.16 | 0.55±0.18 | 0.61±0.11 |
| **T2** | 1.37±0.2 | 1.0±0.2 | 0.44±0.12 | 0.41±0.10 | 0.88±0.18 | 0.81±0.11 | 0.53±0.14 | 0.6±0.21 |
| **T3** | 1.27±0.1 | 1.3±0.4 | 0.43±0.12 | 0.4±0.12 | 0.89±0.16 | 0.82±0.19 | 0.54±0.10 | 0.62±0.19 |
| ***P*-value** | **0.0019*** | 0.1455 | 0.9814 | 0.9859 | 0.9304 | 0.9762 | 0.9954 | 0.9797 |
|  | **Non- Cleft Group-Severe (group Id)** | | | | **Cleft Group-Severe (group IId)** | | | |
| **T0** | 0.62±0.1 | 1.1±0.2 | 0.51±0.21 | 0.48±0.25 | 0.59±0.22 | 0.62±0.32 | 0.46±0.19 | 0.51±0.20 |
| **T1** | 1.3±0.4 | 1.4±0.6 | 0.54±0.11 | 0.52±0.19 | 0.57±0.10 | 0.65±0.21 | 0.51±0.21 | 0.45±0.12 |
| **T2** | 1.1±0.2 | 1.0±0.2 | 0.55±0.09 | 0.5±0.15 | 0.51±0.19 | 0.64±0.20 | 0.5±0.27 | 0.49±0.12 |
| **T3** | 1.2±0.1 | 1.3±0.4 | 0.53±0.18 | 0.51±0.14 | 0.5±0.17 | 0.62±0.23 | 0.53±0.19 | 0.56±0.18 |
| ***P*-value** | 0.1394 | 0.7344 | 0.9955 | 0.9966 | 0.9000 | 0.9984 | 0.9818 | 0.8606 |

**Table S15**. Multiple Comparison *P* Values using Tukey HSD post hoc test for middle ear volumes for group Ia.

| **Intragroup comparison** | **Mean difference** | | | **95 % CI** | | ***P* value** | |
| --- | --- | --- | --- | --- | --- | --- | --- |
|  | **RE** | | **LE** | **RE** | **LE** | **RE** | **LE** |
| **To vs T1** | 0.4000 | 0.3000 | | -0.1143 to 0.6857 | -0.1427 to 0.7427 | **0.0030*** | 0.2807 |
| **To vs T2** | 0.2000 | 0.1000 | | -0.0857 to 0.4857 | -0.3427 to 0.5427 | 0.2544 | 0.9297 |
| **To vs T3** | 0.3000 | 0.2000 | | 0.0143 to 0.5857 | -0.2427 to 0.6427 | **0.0364*** | 0.6236 |
| **T1 vs T2** | -0.2000 | 0.2000 | | -0.4857 to 0.0857 | -0.6427 to 0.2427 | **0.0354*** | 0.6236 |
| **T1 vs T3** | -0.1000 | 0.1000 | | -0.3857 to 0.1857 | -0.5427 to 0.3427 | 0.9982 | 0.9297 |
| **T2 vs T3** | 0.1000 | 0.1000 | | -0.1857 to 0.3857 | -0.3427 to 0.5427 | 0.7846 | 0.9297 |

**Table S16.** Results of Tukey HSD post hoc test of middle ear volume for group IIa.

| **Intragroup comparison** | **Mean difference** | | | **95 % CI** | | ***P* value** | |
| --- | --- | --- | --- | --- | --- | --- | --- |
|  | **RE** | | **LE** | **RE** | **LE** | **RE** | **LE** |
| **To vs T1** | 0.1600 | 0.1300 | | - 0.0402 to 0.3602 | -0.0522 to 0.3122 | 0.1562 | 0.2370 |
| **To vs T2** | 0.1900 | 0.1400 | | - 0.0102 to 0.3902 | - 0.0422 to 0.3222 | 0.0680 | 0.1824 |
| **To vs T3** | 0.1700 | 0.1300 | | - 0.0302 to 0.3702 | - 0.0522 to 0.3122 | 0.1200 | 0.2370 |
| **T1 vs T2** | 0.0300 | 0.0100 | | - 0.1702 to 0.2302 | - 0.1722 to 0.1922 | 0.9774 | 0.9988 |
| **T1 vs T3** | 0.0100 | 0.0000 | | - 0.1902 to 0.2102 | - 0.1822 to 0.1822, | **0.0000*** | 0.9991 |
| **T2 vs T3** | -0.0200 | -0.0100 | | - 0.2202 to 0.1802 | - 0.1922 to 0.1722 | 0.9931 | 0.9988 |

**Table S17**. Results of Tukey HSD post hoc test for middle ear volume for group Ib.

| **Intragroup comparison** | **Mean Difference** | | | **95 % CI** | | ***P* value** | |
| --- | --- | --- | --- | --- | --- | --- | --- |
|  | **RE** | | **LE** | **RE** | **LE** | **RE** | **LE** |
| **To vs T1** | 0.5100 | 0.2900 | | 0.1687 to 0.8513 | -0.0406 to 0.6206 | **0.0018*** | 0.1013 |
| **To vs T2** | 0.3000 | 0.1100 | | -0.0413 to 0.6413 | -0.4406 to 0.2206 | 0.1003 | 0.8005 |
| **To vs T3** | 0.4000 | 0.1900 | | 0.0587 to 0.7413 | -0.1406 to 0.5206 | **0.0169*** | 0.4119 |
| **T1 vs T2** | 0.2100 | 0.4000 | | -0.5513 to 0.2313 | -0.7306 to -0.0694 | 0.3527 | **0.0132*** |
| **T1 vs T3** | 0.1100 | 0.1000 | | -0.4513 to 0.2313 | -0.4306 to 0.2306 | 0.8151 | 0.8418 |
| **T2 vs T3** | 0.1000 | 0.3000 | | -0.2413 to 0.4413 | -0.0306 to 0.6306 | 0.8538 | 0.0857 |

**Table S18**. Results of Tukey HSD post hoc test for middle ear volume for group IIb.

| **Intragroup comparison** | **Mean Difference** | | | **95 % CI** | | ***P* value** | |
| --- | --- | --- | --- | --- | --- | --- | --- |
|  | **RE** | | **LE** | **RE** | **LE** | **RE** | **LE** |
| **To vs T1** | 0.2300 | 0.2200 | | 0.0383 to 0.4217 | 0.0295 to 0.4105 | **0.0141*** | **0.0189*** |
| **To vs T2** | 0.3000 | 0.2300 | | 0.1083 to 0.4917 | 0.0395 to 0.425 | **0.0011*** | **0.0134*** |
| **To vs T3** | 0.3200 | 0.2500 | | 0.1283 to 0.5117 | 0.0595 to 0.4405 | **0.0005*** | **0.0066*** |
| **T1 vs T2** | 0.0700 | 0.0100 | | 0.1217 to 0.2617 | -0.1805 to 0.2005 | 0.7524 | 0.9989 |
| **T1 vs T3** | 0.0900 | 0.0300 | | 0.1017 to 0.2817 | -0.1605 to 0.2205 | 0.5818 | 0.9728 |
| **T2 vs T3** | 0.0200 | 0.0200 | | 0.717 to 0.2117 | -0.1705 to 0.2105 | 0.9918 | 0.9916 |

**Table S19**. Results of Tukey HSD post hoc test for middle ear volume group Ic.

| **Intragroup comparison** | **Mean Difference** | | | **95 % CI** | | ***P* value** | |
| --- | --- | --- | --- | --- | --- | --- | --- |
|  | **RE** | | **LE** | **RE** | **LE** | **RE** | **LE** |
| **To vs T1** | 0.7400 | 0.5300 | | 0.2125 to 1.2675 | -0.1708 to 1.2308 | **0.0050*** | 0.1757 |
| **To vs T2** | 0.7700 | 0.1300 | | 0.2425 to 1.2975 | -0.5708 to 0.8308 | **0.0036*** | 0.9503 |
| **To vs T3** | 0.6700 | 0.4300 | | 0.1425 to 1.1975 | -0.2708 to 1.1308 | **0.0108*** | 0.3294 |
| **T1 vs T2** | 0.0300 | -0.4000 | | 0.4975 to 0.5575 | -1.1008 to 0.3008 | 0.9984 | 0.3891 |
| **T1 vs T3** | 0.0700 | -0.1000 | | 0.5975 to 0.4575 | -0.8008 to 0.6008 | 0.9807 | 0.9762 |
| **T2 vs T3** | 0.1000 | 0.3000 | | 0.6275 to 0.4275 | -0.4008 to 1.0008 | 0.9473 | 0.6208 |
